# Combining the Vaginal Microbiome and Serum Metabolome to Screen for Potential Biomarkers of Early Pregnancy in Cows

**DOI:** 10.3390/metabo14090469

**Published:** 2024-08-26

**Authors:** Yan Luo, Zhen Wang, Xin Zhao, Jiankang Xing, Zhiliang Chen, Wenxue Zhao, Xiaoqing Long, Yanbing Zhang, Yongbin Shao

**Affiliations:** College of Animal Science and Technology, Shihezi University, Shihezi 832003, China; luoyan@shzu.edu.cn (Y.L.); 19839912829@163.com (Z.W.); 18167531767@139.com (X.Z.); 18240930359@163.com (J.X.); 18888501796@163.com (Z.C.); 18363875632@139.com (W.Z.); 18099763763@163.com (X.L.); zhangyanbing@shzu.edu.cn (Y.Z.)

**Keywords:** early pregnancy, metabolomics, flora diversity, biomarkers, arachidonic acid metabolic pathway

## Abstract

Early pregnancy diagnostic techniques are of significant importance in livestock farming, particularly in dairy farming. This study aimed to screen artificially inseminated cows for potential biomarkers at day 21 of pregnancy using microbiota–metabolomics analysis. The microbiome analysis revealed significant changes (*p* < 0.05) in the composition and abundance of the vaginal microbiota in cows after pregnancy. Notably, there was an increase in the abundance of *[Eubacterium]_hallii_group* (*p* < 0.05) associated with the production of short-chain fatty acids in the pregnant group compared with the non-pregnant group. Furthermore, significant alterations were observed in the serum metabolome, with notable changes in the concentrations of prolyl-hydroxyproline (Pro-Hyp) (*p* < 0.01) and bonactin (*p* < 0.01). The majority of differential metabolites clustered within the pathways of amino acid metabolism and lipid metabolism, with lipid metabolism exhibiting a higher proportion and playing a pivotal role in early pregnancy. An enzyme-linked immunosorbent assay was employed to quantify three key metabolites of the arachidonic acid pathway. The results demonstrated significant decreases in serum concentrations of leukotriene B4 (LTB4) (*p* < 0.05) and prostaglandin F2α (PGF2α) (*p* < 0.01) and no significant changes in arachidonic acid (AA) (NS) concentrations after 21 days of gestation in cows. Spearman’s correlation analysis was utilized to investigate the interrelationship between the vaginal microbiota and serum metabolites. In conclusion, the present study demonstrated that biomaterials such as bonactin, Pro-hyp, LTB4, PGF2α in serum metabolites and *[Eubacterium]_hallii_group* in the vaginal flora of cows could be utilized as potential biomarkers for 21 days of gestation in cows.

## 1. Introduction

A delay in the identification of early pregnancy can result in an extended breeding period in the subsequent lactation, an increased calving interval, a reduction in fertility, and higher breeding costs. The early detection of pregnancy following the artificial insemination of cows is of significant importance, as it allows for the protection of the fetus and the enhancement of the pregnancy rate. Consequently, the early diagnosis of pregnancy is crucial [1].

The conventional approach to pregnancy diagnosis entails evaluating a series of changes in the cow’s physiological functions and related biochemical indices following conception. The rectal palpation technique represents the most commonly employed methodology for diagnosing pregnancy in cows. This procedure involves detecting morphological changes in the uterine horns, corpus luteum, amniotic sac, and fetal structures through the rectal wall [2]. Similarly, ultrasonography is an invaluable tool in the early detection of pregnancy in cows. It is employed to diagnose the pregnancy status of the cow by evaluating the development of the follicle and the fetus in accordance with the characteristics of ultrasonic imaging of various parts of the cow’s body [3]. Nevertheless, this technique necessitates the utilization of more costly equipment and the possession of the requisite knowledge of ultrasound imaging by the operator. During the initial stages of pregnancy, the corpus luteum and embryo of the cow produce substantial quantities of progesterone, which can be quantified in the blood [4]. However, it has been demonstrated that a prolonged luteal phase in some non-pregnant cows results in a higher incidence of false positives in progesterone testing, which has a significant impact on the accuracy of the results [5]. At present, the best method for detecting pregnancy in cows is the use of pregnancy-associated glycoproteins (PAGs). It has been shown that as early as the 15th day after conception, secretions from the PAG are present in the circulation of bovine dams [6]. However, the highest sensitivity and specificity of pregnancy detection by PAG occurs between 26 and 32 days after insemination of the cow [7,8]. Importantly, a cow’s estrous cycle is typically 21 days. Due to the temporal limitations of the above-mentioned methods of pregnancy diagnosis, it can lead to a missed mating period for cows that do not become pregnant after artificial insemination, which ultimately increases the cost of reproduction [9]. Consequently, the identification of biomarkers at 21 days of gestation in cows is essential to enhance the precision of pregnancy detection and to facilitate the earlier recognition of pregnancy.

Metabolomics, a method utilizing various biological samples such as urine, feces, blood, and tissues, offers a deeper understanding of the body’s metabolic reactions to external factors [10]. An in-depth exploration of small molecule metabolites in biological systems can be carried out at precise time points and under well-defined conditions [11]. Recently, metabolomic approaches have been utilized to explore novel targets and pathways in diagnosing illnesses in dairy cattle, understanding the physiology of cows, and assessing the fertility of these animals [12]. Notably, alterations in the composition of microorganisms and their metabolites are closely associated with the onset and regulation of disease [13]. Zhai and colleagues utilized metabolomic and proteomic methodologies to detect serum amyloid A (SAA) and fetal antigen marker (FAM) in the serum of pregnant ewes. Their objective was to evaluate the feasibility of using these biomarkers as indicators of early pregnancy status in ewes [14]. The vaginal microflora is a complex and heterogeneous group of organisms consisting of various species and strains. Their numbers and proportions vary considerably between individuals and over time. Typically, the vaginal microbiota reflects the physiological status of the female, both quantitatively and qualitatively. This can stimulate the release of metabolites from the host, which can subsequently impact the immune system, bloodstream, and circulatory system [15]. Zhang and collaborators noted that the vaginal flora plays a role in the synthesis of sex hormones, affects serum metabolic indices, and contributes to the resumption of estrus in sows post-weaning [16]. Furthermore, the vaginal microbiome of cows can be employed as a biomarker of cow reproduction, with the capacity to predict reproductive potential and gestational competence [17].

This study aimed to investigate the vaginal flora and serum metabolites of cows on the 21st day post-artificial insemination using 16S rDNA sequencing and serum metabolomics. Additionally, the study aimed to uncover connections between vaginal flora and serum metabolites, as well as identify potential biomarkers for early pregnancy detection in cows.

## 2. Materials and Methods

### 2.1. Experimental Animals and Groups

A set of experiments was carried out on a group of Holstein cows, with an average body weight of 550 ± 50 kg and a similar mean body condition score, sourced from a cattle farm in Shihezi Xinjiang. These cows had experienced two or three pregnancies and exhibited typical growth and development patterns. They displayed moderate fattening and appeared to be in good overall health. The cows are fed a total mixed ration (TMR) by the breeder. And the dry matter intake is around 18.5 kg per day. The cows have free access to food, water, and exercise on the farm. The cows are milked normally every day.

A random subset of 60 cows underwent a 21-day period of artificial insemination. In order to exclude the influence of exogenous hormones on the serum metabolites, all the cows were brought into estrus under natural conditions. The cows’ return to estrus was monitored until 28 days post-insemination. An ultrasound scan was then performed on day 32 of insemination to determine early pregnancy status. Based on the scan results, the cows were categorized into the “pregnant group” (P) if they were pregnant and the “non-pregnant group” (NP) if they were not pregnant.

### 2.2. Collection and Preparation of Samples

On the 21st day after the insemination procedure, blood samples were collected from the cows through venipuncture of their caudal veins. The serum was then separated from the blood by static standing at 4 °C, after which it was frozen at −80 °C for long-term storage. The cow’s vulva was cleaned with water and disinfected using a 25% iodine solution. A cotton swab was inserted into two-thirds of the vagina for one minute to ensure full contact with vaginal secretions. The swab was then placed in a 1.5 mL centrifuge tube and promptly frozen in liquid nitrogen for preservation. Subsequently, six serum samples from cows were randomly chosen from both the pregnant (P) and non-pregnant (NP) groups for non-targeted metabolome analysis. Additionally, three vaginal swab samples from each group were randomly selected for analysis of vaginal flora diversity.

### 2.3. Vaginal Flora Diversity Analysis

#### 2.3.1. 16S rDNA Gene Sequencing Analysis

The genomic DNA from the vaginal flora was extracted using the cetyltrimethylammonium bromide (CTAB)/sodium dodecyl sulfate (SDS) method. After extraction, the purity and concentration of the DNA were assessed through 1% agarose gel electrophoresis. The DNA was then diluted to a concentration of 1 ng/μL with sterile water and used as a template for amplifying the 16S rDNA gene using the specific primer 515F806R (V3–V4). The PCR reaction included 15 µL of Phusion^®^ High-Fidelity PCR Master Mix (New England Biolabs, Ipswich, MA, USA) under optimized conditions, with a primer concentration of 2 µM and 10 ng of genomic DNA template. The PCR products were verified by electrophoresis on a 2% agarose gel, and the successful products were purified using magnetic beads, quantified enzymatically, and combined in equal amounts based on their concentrations. The resulting mixture underwent another round of electrophoresis on a 2% agarose gel, and the target bands were isolated using the Universal DNA Purification and Recovery Kit (Tian Gen, Beijing, China, Catalog#: DP214).

The library construction was carried out using the NEB Next Ultra II FS DNA PCR-free Library Prep Kit from New England Biolabs (Waltham, MA, USA; catalog number: E7430L). The quantity of the constructed libraries was assessed through quantification using both the Qubit and Q-PCR assays. After library construction, the libraries were quantified using Qubit and Q-PCR methods and then subjected to paired-end (PE) 250 sequencing on a NovaSeq 6000 platform.

#### 2.3.2. Statistical Analysis of 16S rDNA Vaginal Flora

Small fragment libraries were specifically constructed to align with the amplified regions, and these libraries underwent paired-end sequencing on the Illumina NovaSeq platform. The generated data underwent processing using tools such as Python (Version 3.6.13) to split and filter the sequence reads for each set of samples. Software such as QIIME2 (Version QIIME2-202202) was utilized for ASV clustering and species annotation, with clustering set at a default similarity level of 97%. Subsequent analyses included alpha diversity and community structure assessments. Alpha diversity was evaluated using indices such as richness (Chao, ACE), diversity (Simpson, Shannon), and coverage (Good’s coverage). To further investigate differences in community structure between groups, LEfSe was employed to determine the statistical significance of variations in species composition and community structure across the samples.

The data were analyzed utilizing a two-sample *t*-test in R (Version 4.0.3). Significance testing was conducted at a level of *p* < 0.05, with results presented as *p*-values. A metastat analysis was carried out using R (Version 4.0.3) software, with significance testing set at a *p*-value of <0.05. LEfSe analyses were executed on the NovoCloud platform (https://magic.novogene.com/, accessed on 8 November 2023), with significance assessed using an LDA Score of >3 and a *p*-value of <0.05. The outcomes from these analyses were represented as *p*-values.

### 2.4. Serum Untargeted Metabolomics Sample Preparation and Analysis

#### 2.4.1. Sample Preparation

The process begins by removing the samples from the −80 °C refrigerator and ensuring they thaw slowly to maintain sample integrity. Once thawed, an appropriate amount is extracted using a solution of pre-cooled methanol, acetonitrile, and water in a 2:2:1 ratio. The samples are then vortexed, subjected to low-temperature ultrasound, and cooled to −20 °C for 10 min. Subsequently, centrifugation at 14,000× *g* at 4 °C for 20 min separates the supernatant, which is then dried in a vacuum. For mass spectrometry, 100 μL of acetonitrile is added, and the sample is prepared by mixing water in a 1:1 (*v*/*v*) ratio, vortexing, and centrifuging at 14,000× *g* for 15 min at 4 °C.

The preparation of quality control (QC) samples involves transferring all serum samples to a 96-well microtiter plate using a pipette. The samples are then mixed by vortexing for 2 min to ensure homogeneity and prepare QC samples containing all serum samples. Following a consistent and standardized procedure for both QC and individual serum samples is essential for maintaining the quality and accuracy of the analytical results.

#### 2.4.2. Chromatography-Mass Spectrometry (MS)

The samples were separated on a Vanquish LC ultra-high performance liquid chromatography (UPLC) system with a HILIC column; the column temperature was 25 °C; the flow rate was 0.3 mL/min; the injection volume was 2 μL; the mobile phases were A: water +25 mM ammonium acetate +25 mM ammonia and B: acetonitrile; and the gradient elution procedure was as follows: 0–1.5 min, 98% B; 1.5–12 min, B varied from 98% to 2%; 12–14 min, B held at 2%; 14–14.1 min, B varied linearly from 2% to 98%; 14.1–17 min, B held at 98%; the sample was placed in the autosampler at 4 °C throughout the analysis. To avoid the effects of variations in the instrumental detection signal, samples were analyzed continuously in random order. QC samples were included in the sample queue to monitor and evaluate the stability of the system and the reliability of the experimental data.

The samples were analyzed using a Q-Exactive series mass spectrometer for both primary and secondary spectra. Prior to mass spectrometry analysis, the samples were separated using a Vanquish LC ultra-high performance liquid chromatography (UPLC) system. The mass spectrometry analysis was conducted with the Q-Exactive series mass spectrometer from Thermo Fisher Scientific (Waltham, MA, USA) using electrospray ionization (ESI) in both positive and negative modes. The ESI source and mass spectrometry parameters were set as follows: Nebulizing gas, Auxiliary heating gas 1 (Gas1) 60, Auxiliary heating gas 2 (Gas2): 60, curtain gas (CUR): 30 psi, ion source temperature: 600 °C, spray voltage (ISVF) ± 5500 V (positive and negative modes); detection range of the first level of mass-to-charge ratio: 80–1200 Da, resolution: 60,000, scan accumulation time: 100 ms, detection range of the first level of the mass-to-charge ratio: 80–1200 Da, resolution: 60,000 and detection range of first level of mass to charge ratio: 80–1200 Da, resolution: 60,000. Scanning accumulation time: 100 ms, the second level adopts segmented detection method, the scanning range is 70–1200 Da, the resolution of the second level: 30,000, scanning accumulation time: 50 ms, dynamic exclusion time: 4 s.

#### 2.4.3. Serum Metabolomics Data Processing and Statistical Analysis

The raw data obtained from the mass spectrometry analysis were converted to mzXML format using ProteoWizard (version 3.0.4146).

Subsequently, software (http://xcmsonline.scripps.edu, accessed on 10 October 2023) was utilized for tasks such as peak alignment, retention time correction, and peak area extraction. Metabolites underwent further analysis, including structural characterization through precise mass matching (<25 ppm) and secondary spectral matching, involving searches in laboratory databases.

For statistical analysis and data interpretation, the SIMCA P+ 14.1 software from Umetrics in Umea, Sweden, was employed. Multivariate analyses, including Principal Component Analysis (PCA) and Orthogonal Partial Least Squares Discriminant Analysis (OPLS-DA), were carried out. The model was evaluated for stability using sevenfold cross-validation and response replacement tests. Variable importance (VIP) values within the OPLS-DA model were used to assess the contribution of each variable to classification.

Further steps involved querying precise relative molecular masses in HMDB and KEGG public databases to establish databases of the chemical compositions of the metabolites. Biomarker identification was performed in conjunction with literature references and spectroscopic information. Correlation analyses of metabolic pathways were conducted using MetaboAnalyst.

The data obtained underwent statistical analyses using SPSS 26.0 software [18], presenting the results as mean ± standard deviation ($\bar{x}\pm s$). The statistical analysis involved comparing data between groups through a one-way ANOVA. Metabolites with Variable Importance in Projection (VIP) values exceeding one were further analyzed using a univariate level Student’s *t*-test to assess their significance. A significance level of *p* < 0.05 was established to indicate a statistically significant difference between groups.

### 2.5. Metabolic Pathway Marker Validation

The key differential metabolites, including LTB4, PGF2α, and AA, were selected based on histological results. The levels of these relevant metabolites were measured in the sera of cows from both the non-pregnant (NP) group and the pregnant (P) group. The detection was carried out following the protocols outlined in the ELISA kits instruction manual obtained from Shanghai Enzymede-Link Biotechnology Co., Ltd. (Shanghai, China).

### 2.6. Histological Correlation Analysis

The differential metabolites associated with the differential microorganisms were identified through correlation analysis, with Spearman correlation coefficients calculated. Metabolites were identified as potentially affected by microorganisms if they exhibited a correlation coefficient of r ≥ 0.6 or r ≤ −0.6, in conjunction with a Benjamini and Hochberg (BH) corrected *p*-value of <0.05. The differences were also corrected for multiple hypothesis testing using the BH method. This method controls for false discovery rate (FDR) and thus considers differences statistically significant at *p* < 0.05.

## 3. Results

### 3.1. Analysis of Vaginal Flora Diversity

#### 3.1.1. Abundance Composition of the Vaginal Flora and Diversity of Vaginal Microorganisms

In this study, a total of 897,788 valid sequences were obtained from the two groups, with an average length of 414.77 base pairs. Subsequently, after applying various filters to eliminate low-quality data, sequences underwent noise reduction, double-ended sequence splicing, and removal of chimeric elements. These processes led to the identification of 640,389 highly accurate sequences for further analysis.

To assess microbial richness and diversity and characterize microbial α-diversity, we employed the Chao, Simpson, and Shannon indices. Analysis of the data revealed that there were no statistically significant variations in the Chao1, Shannon, and Simpson indices between the two groups (Table 1). These results suggest that there were no significant differences in microbial community diversity or changes in colony abundance, indicating the similarity in vaginal flora composition between the non-pregnant and pregnant cows groups.

**Figure 1:**
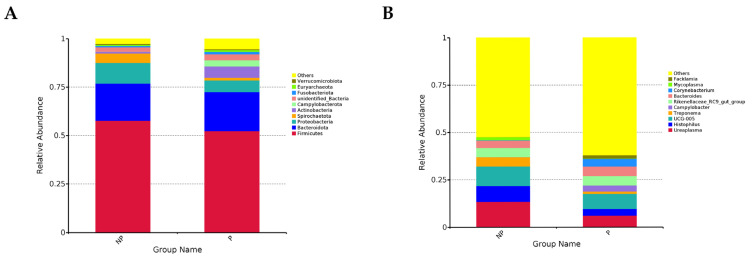
Composition of the vaginal microbiota at the phylum and genus levels in the pregnant and non-pregnant groups. (**A**) The top 10 phyla with the highest abundance in each group. (**B**) Top 10 genera with the highest abundance in each group.

In the analysis presented in Figure 1A,B, the relative abundance of the top 10 bacteria at the phylum and genus levels was examined to identify variations in the abundance of major dominant phyla and genera. At the phylum level, there was a decrease in the relative abundance of Sclerotinia, Aspergillus, and Spirochaetes from 57.7%, 10.6%, and 5.0% to 52.3%, 6.0%, and 1.4%, respectively. On the other hand, the proportion of the phylum Anabaena increased from 19.3% to 20.2%, while that of the phylum remained relatively stable. These observed changes were not found to be statistically significant. At the genus level, the percentages of *Ureaplasma*, *UCG-005*, and *Histophilus* decreased from 13.5%, 10.4%, and 8.3% to 6.1%, 7.9%, and 3.6%, respectively. In contrast, the relative abundance of *Campylobacter* rose from 0.3% to 3%.

#### 3.1.2. Vaginal Microbiota Analysis and Functional Prediction

**Figure 2:**
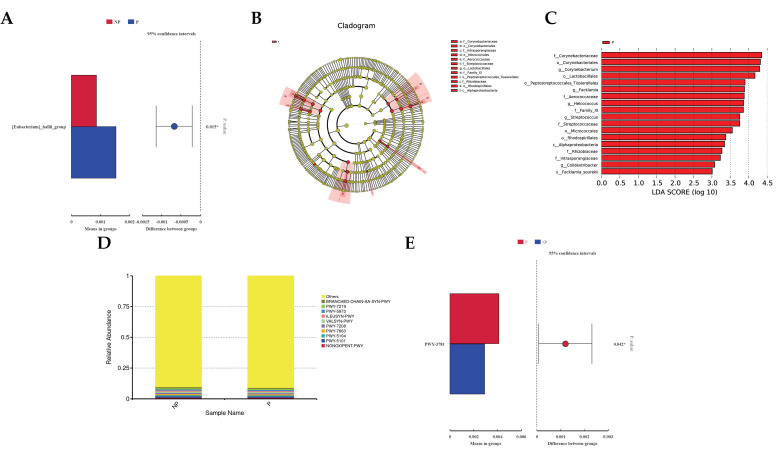
Differential analysis of microbiota as well as functional prediction. (**A**) Differences in the genus level of the bacterial flora in the pregnant and non-pregnant groups were analyzed using STAMP. (**B**) LEfSe analysis of the phylogenetic distribution of vaginal microbiota from phylum to genus in each group. (**C**) Evolutionary maps generated by the LEfSe LDA analysis identifying bacterial abundance (LDA Core ≥ 3) between the two groups. (**D**) KEGG metabolic pathways of the statistical plot PICRUSt2 predicted bacterial pathway abundance map. (**E**) STAMP analysis of differences in bacterial metabolic pathways between the pregnant and non-pregnant groups. * *p* < 0.05.

The LEfSe method was utilized to identify specific differences in bacterial enrichment between the pregnant and non-pregnant groups. Across phylum to genus levels, a total of 17 differential features were detected (refer to Figure 2C), and the abundance of *Corynebacterium*, *Facklamia*, *Helcococcus*, *Streptococcus*, and *Colidextribacter* was significantly higher in the pregnant group compared to the non-pregnant group (LDA score > 3.0, *p* < 0.05). Notably, the abundance of flora (LDA Core ≥ 3) differed significantly between the groups. Additionally, STAMP analysis highlighted a significant variance in the *[Eubacterium]_hallii_group* between the two groups (*p* < 0.05).

Furthermore, PICRUSt2 analysis was conducted to assess the metabolic pathway abundance of vaginal flora based on 16S sequencing data. The top 10 pathways in terms of abundance were presented in Figure 2D, with predicted pathways such as NONOXIPENT-PWY, PWY-5101, PWY-5104, PWY-7663, PWY-7208, VALSYN-PWY, ILEUSYN-PWY, PWY-5973, PWY-7219, and BRANCHED-CHAIN-AA-SYN-PWY. Among these pathways, isoleucine synthesis, adenosine ribonucleotide 9 biosynthesis, pentose phosphate pathway, super pathway of pyrimidine nucleobase recycling, and valine synthesis showed no significant differences, whereas PWY-3781 exhibited a significant difference (*p* < 0.05), indicating a correlation with cellular aerobic respiration (Figure 2E).

### 3.2. Serum Untargeted Metabolomics Analysis

#### 3.2.1. Multivariate Statistical Analyses

The sample quality control (QC) analysis revealed that the response intensities and retention times of the Total Ion Chromatogram (TIC) peaks largely overlapped, as depicted in Figure S1A–H. Over 80% of the peaks in the QC samples exhibited Relative Standard Deviation (RSD) ≤ 30%, indicating good stability in the instrumental analysis system. Principal Component Analysis (PCA) was employed to compare serum samples from the Non-Pregnant (NP) group with those from the Pregnant (P) group. In the positive ion mode (Figure S1A–H), R2X = 0.551, which is greater than 0.4, and in the negative ion mode (Figure S1A–H), R2X = 0.561, also exceeding 0.4. The NP, P, and QC groups showed aggregated distributions with more concentrated patterns. The Orthogonal Partial Least Squares Discriminant Analysis (OPLS-DA) model indicated effective clustering of the two sample groups, highlighting a clear distinction between them and a reliable predictive model. Differences in serum metabolite abundance between the NP and P groups in positive and negative ion modes are shown in Figure S1A–H (R2 Y = 92.3%, Q2 = 11.2%) and Figure S1A–H (R2 Y = 90.7%, Q2 = 6.36%), respectively. The high intra-group aggregation and significant inter-group separation further underline the reliability of the model, showcasing substantial differences between the groups.

Moreover, as the replacement retention decreases (refer to Figure S1A–H), both R2 and Q2 of the stochastic model also decrease gradually without overfitting, indicating robustness in the original model.

#### 3.2.2. Serum Metabolomics Analysis

**Figure 3:**
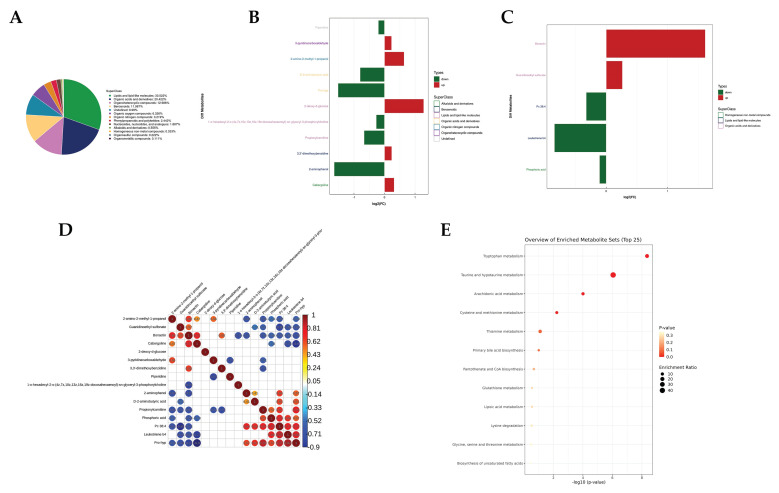
Analysis of serum differential metabolites in cows. (**A**) Hierarchical clustering analysis of differential metabolites. (**B**) FC values of differential metabolites and metabolic clustering in positive ion mode. (**C**) FC values of differential metabolites and metabolic clustering in negative ion mode. Note: Red represents an increase, and green represents a decrease. (**D**) Metabolite correlation with significant difference between two groups. (**E**) Pathway enrichment map of differential metabolites.

In the serum metabolomics analysis, a total of 901 metabolites were identified, with 468 metabolites detected in the positive ion mode and 433 metabolites in the negative ion mode. These identified metabolites were predominantly lipids and lipid-like molecules (30.522%), followed by organic acids and derivatives (20.422%), organoheterocyclic compounds (12.986%), and benzenoids (11.987%). This suggests that lipid metabolism, along with some organic and amino acid metabolism, was prominent during the early gestation period of the cows, as illustrated in Figure 3A.

Significant alterations were observed in the serum metabolites of cows following pregnancy, leading to the identification of 16 potential metabolic biomarkers (VIP > 1.000, *p* < 0.050, AUC > 0.800), as presented in Table 2. Among these biomarkers, seven metabolites exhibited significantly higher relative concentrations, while nine metabolites showed significantly lower relative concentrations in the serum of pregnant cows. The differential metabolites were mainly comprised of lipids and lipid-like molecules and organic acids and derivatives under positive and negative ions, illustrated in Figure 3B,C.

Correlation analysis of the metabolites revealed interactions and strong correlations between the metabolites among them, with examples such as Pro-hyp displaying a negative correlation with cabergoline (r = −0.9) and a positive correlation with LTB4 (r = −0.82). Additionally, Pc 38:4 was negatively correlated with guanidinoethyl sulphonate (r = −0.85), as depicted in Figure 3D.

Furthermore, enrichment analysis of the differential metabolites indicated that most of these metabolites were enriched in pathways related to tryptophan, taurine, hypotaurine, arachidonic acid, cysteine and methionine, and thiamine (*p* < 0.01 and enrichment ratio ≥ 10), as shown in Figure 3E.

### 3.3. Metabolic Pathway Marker Validation

**Figure 4:**
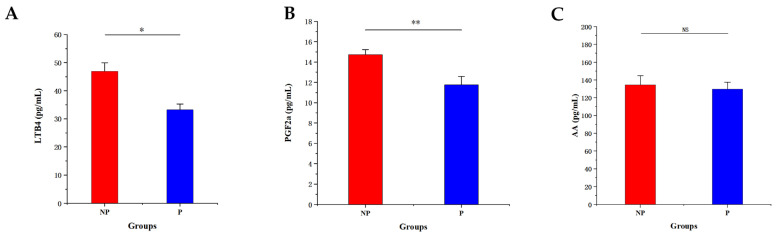
Graph of metabolite concentrations by ELISA. (**A**) Concentration of LTB4 in the serum of cows. (**B**) Concentration of PGF2α in the serum of cows. (**C**) Concentration of AA in the serum of cows. Note: NP, non-pregnant group; P, pregnant group. Comparison with NP * *p* < 0.05; ** *p* < 0.01, NS means no significant difference.

The concentrations of LTB4, PGF2α, and AA in the serum of cows in the non-pregnant and pregnant groups were examined by ELISA, and it was found that, compared with the non-pregnant group, there was a significant decrease in the concentration of LTB4 in the serum metabolites of cows in the pregnant group (*p* < 0.05), a highly significant decrease in the concentration of PGF2α (*p* < 0.01) and a non-significant difference in the change in the concentration of arachidonic acid, AA, in the pregnant group (NS) (Figure 4).

### 3.4. Spearman Correlation Analysis

**Figure 5:**
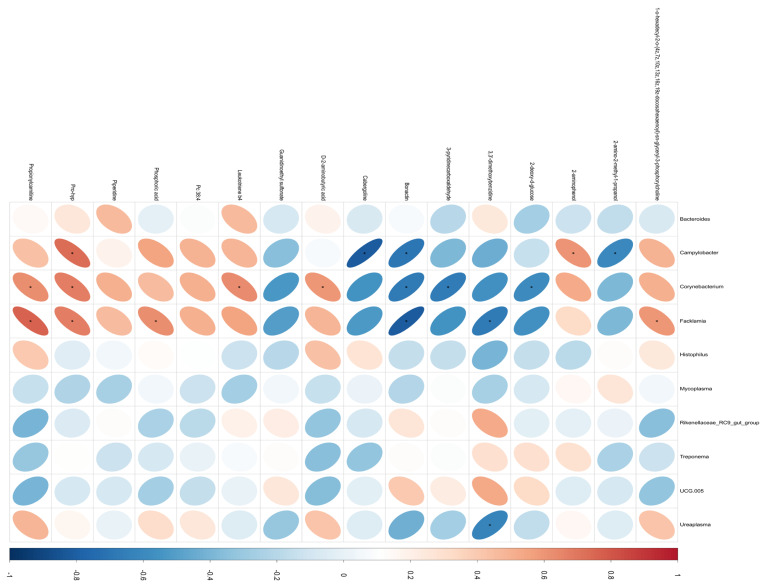
Heat map of correlation between vaginal microbiota and serum metabolites. * *p*-value < 0.05. The color blue is used to represent a negative correlation, whereas the color red is used to represent a positive correlation.

The correlation between the differential metabolites in bovine serum and the abundance of the top 10 vaginal flora at the genus level was analyzed through a correlation heatmap, as shown in Figure 5 Spearman’s correlation coefficient (r) and the corresponding *p*-value were used for this analysis, where correlations with |r| ≥ 0.6 and *p* < 0.05 were considered statistically significant.

A total of 18 pairs of significantly related microbiota and metabolites were identified, with 9 pairs showing significant positive correlations and 9 pairs displaying significant negative correlations. For example, the relative abundance of *Campylobacter*, *Facklamia*, and *Corynebacterium* was found to be negatively correlated with the serum differential metabolite bonactin (*p* < 0.05) and positively correlated with Pro-hyp (*p* < 0.05). Conversely, the relative abundance of these genera was negatively correlated with serum differential metabolites such as 3,3′-dimethoxybenzidine, 3-pyridinecarboxaldehyde, and cabergoline, while being positively correlated with LTB4, phosphoric acid, and propionylcarnitine (*p* < 0.05).

Furthermore, it was observed that the relative abundance of both *Corynebacterium* and *Facklamia* was significantly increased in cows post-pregnancy (LDA Score ≥ 3). This elevation in abundance was suggested to be associated with the development of inflammation based on relevant studies, highlighting a potential link between the vaginal flora composition and systemic metabolic changes following pregnancy in cows.

## 4. Discussion

Accurate and timely diagnosis of pregnancy is crucial for the cattle industry. This study aimed to explore the variations in vaginal microbiota and serum metabolite levels in non-pregnant cows compared to cows at 21 days of gestation to identify potential biomarkers of pregnancy in cows. The integration of new research technologies has facilitated a deeper understanding of the connection between microbiota, metabolomics, health, and disease. It is crucial to acknowledge that the vaginal flora of women undergoes a dynamic transformation throughout the course of pregnancy and childbirth [19]. Furthermore, alterations in vaginal flora not only influence the outcome of pregnancy but also have an impact on women’s general health [20]; the vaginal flora plays a significant role in the course of pregnancy in women. Recent evidence suggests that alterations in the vaginal microbiota are linked to pregnancy outcomes and changes during pregnancy in ewes [21]. Deng et al. investigated the vaginal and fecal microbiota of beef cattle and found significant differences in the diversity of the vaginal microbiota between gestation stages [17]. Additionally, a shift in the diversity and phylogenetic relationship of these diverse microbial communities up to the time of breeding can also lead to a good pregnancy in cattle [22,23,24]. Changes in the vaginal flora of cows in early pregnancy may influence pregnancy outcomes.

Therefore, we investigated alterations in the vaginal microbiota of cows at 21 days of gestation using 16S rDNA gene sequencing. In this study, the relative abundance of microbiota in the vaginal secretions of cows at 21 days of gestation exhibited insignificant variations at the phylum level but more significant differences at the genus level. Previous studies have reported that the most abundant bacterial phyla in the bovine vagina are Firmicutes, Bacteroidetes, and Actinobacteria, respectively [25]. Our results are consistent with previous studies. At the phylum level, Firmicutes and Bacteroidetes are associated with the production of short-chain fatty acids such as butyrate and acetate, which play a role in immunomodulation and contribute to the stabilization of the early embryo [26]. At the genus level, we observed an elevated relative abundance of *Corynebacterium* spp., which can produce ethylene glycol, a pathogenic microbial metabolite linked to preterm labor in women [27]. Additionally, its relative abundance increases with the cow’s litter size [28], potentially compromising stable fetal development.

Notably, we found a significant (*p* < 0.05) increase in the relative abundance of the *[Eubacterium]_hallii_group* in cows at 21 days of gestation. This group correlates with the production of short-chain fatty acids, which can alleviate chronic inflammation and prevent lymphoma to some extent [29]. Furthermore, colony-derived short-chain fatty acids may improve pregnancy outcomes in sows by enhancing ovarian steroidogenesis and endometrial tolerance while limiting vaginal pathogen abundance during early gestation [30]. Increased relative abundance of this genus appears to have a beneficial effect on the cow’s resistance to vaginal pathogens and the progression of pregnancy.

In summary, the 16S rDNA results indicate that the vaginal microbiota of cows in early pregnancy exhibit less variability, with no significant taxonomic differences detected, consistent with the findings of Emily M. Webb et al. [31]. Metabolite changes parallel disruptions in vaginal microbiota during impaired reproductive function onset and progression.

During early gestation, females undergo adjustments in their endocrine system, immune system, and metabolites to facilitate fetal-maternal contact [32]. Simultaneously, the metabolism of many metabolites undergoes changes, as evidenced by significantly higher levels of seven metabolites and lower levels of nine metabolites in cows at 21 days of early pregnancy (Table 2). Among these, changes in metabolites are predominantly dominated by lipids and lipid-like molecules and organic acids and derivatives (Figure 4A).

Among the metabolites with elevated levels, the concentration of bonactin was significantly increased (*p* < 0.01 and FC > 3). Bonactin, a newly isolated compound from liquid cultures of *Streptomyces* spp. [33,34] has been shown to inhibit mycelium growth [35] and spore motility [36] and exhibit antibacterial and antifungal activities, necessitating further investigation into its functions.

It is also noteworthy that Pro-Hyp concentrations were significantly lower (*p* < 0.01) among metabolites with reduced levels. Pro-Hyp, a collagen-derived dipeptide, serves as a new low molecular weight growth initiation factor [37]. In mid-pregnancy, the percentage of collagen degeneration in superficial and deeper matrix layers is significantly higher than in the non-pregnant state [38]. Pro-Hyp metabolic levels can serve as an indicator for collagen content assays. Moreover, Pro-Hyp strongly promotes early chondrocyte differentiation under physiological hypoxic conditions, possibly contributing to fetal growth and differentiation [39].

Based on metabolic pathway analysis, the metabolic pathways where the differential metabolites converged were primarily associated with amino acid metabolism, lipid metabolism, and other functions (Figure 4E). Guo et al. studied plasma metabolism characteristics in artificially inseminated cows after 17 and 45 days of gestation, predominantly dominated by amino acid metabolism and lipid metabolism [40], and the results of the present study were similar to them. Amino acid metabolism has an important role during gestation; as amino acids serve as raw materials for fetal growth and promote cell proliferation. Studies have demonstrated a significant decrease in tryptophan levels in the tryptophan metabolic pathway, along with an increase in indoleamine 2,3-dioxygenase activity during early gestation in cows. These changes may be associated with establishing immune mechanisms for embryonic tolerance [41].

Lipid metabolism in early pregnancy is indeed vital, as it is associated with uterine tolerance and early embryonic development [42]. In our study, lipid substances constituted the highest percentage of detected metabolites. Metabolic pathway analysis revealed a significant change in the lipid metabolic pathway, particularly the arachidonic acid metabolic pathway (*p* < 0.01).

The arachidonic acid pathway plays a crucial role in various physiological processes, including the maintenance of organ function, inflammatory response, drug metabolism, and the development of several diseases, such as cardiovascular disease and cancer. Arachidonic acid (AA) serves as a critical metabolic precursor in this pathway, essential for both the onset and resolution of inflammation. Moreover, arachidonic acid is necessary in early fetal development [43]. In the arachidonic acid metabolic pathway, LTB4 and PGF2α are derived from AA. And they can be rapidly produced from AA [44]. In the present study, the concentration of LTB4, an inflammatory mediator with strong chemotactic and immunomodulatory effects, was significantly reduced (*p* < 0.05) [45]. Jian et al. have suggested that LTB4 may play a crucial role in the success and maintenance of a healthy pregnancy, and it may even have a predictive function for pre-eclampsia in women [46]. Anna J Korzekwa et al. demonstrated that the expression and level of LTB4 receptor mRNA are higher in luteal tissue during pregnancy compared to the estrous cycle, and this is associated with early pregnancy in cattle [47]. PGF2α plays crucial roles in various reproductive aspects such as ovulation, embryogenesis, implantation, and cell proliferation [48]. Studies have indicated that the concentration of PGF2α in the endometrium increases significantly during the first month or less of gestation in cows. PGF2α is essential for maintaining uterine stability in cows during the early stages of pregnancy [49,50]. Piotr Kaczynski and colleagues demonstrated that elevated concentrations of PGF2α in the uterine cavity can stimulate angiogenesis, enabling early gestation embryos in pigs to establish contact with the mother [51]. This suggests that an increase in the concentration of PGF2α in the endometrium during the early stages of pregnancy in animals facilitates the development of early embryos. In this study, it was verified by ELISA that the decrease in serum PGF2α concentration was highly significant (*p* < 0.01). The reduction in serum concentration was associated with an increase in endometrial PGF2α concentration, which primarily promotes embryo implantation and the connection between the embryo and the mother. In conclusion, PGF2α, LTB4, and AA converge in the arachidonic acid pathway and are involved in lipid metabolism associated with early fetal development, which is crucial for early pregnancy in cows.

Spearman’s correlation analysis revealed significant correlations between vaginal microorganisms and several serum metabolites related to lipid metabolism and immunity. The relative abundance of *Corynebacterium* was positively correlated with the concentrations of LTB4 and Pro-Hyp, with LTB4 being associated with inflammation. Additionally, the relative abundance of *Campylobacter* was positively correlated with serum concentrations of 2-aminophenol and Pro-Hyp and negatively correlated with concentrations of cabergoline. Notably, *Corynebacterium* can be isolated from the uterus of cows suffering from endometritis [52], which triggers vagina-associated inflammation in cows, and *Campylobacter* infection triggers the failure of the reproductive organs in ruminants, as well as being the main cause of abortions in sheep [53]. Interestingly, the relative abundances of both *Corynebacterium* and *Campylobacter* were negatively correlated with the concentration of bonactin, which has antimicrobial activity, indicating a correlation between serum metabolites and vaginal flora.

## 5. Conclusions

In conclusion, this study demonstrated a significant reduction in the relative abundance of the vaginal microbiota, specifically the *[Eubacterium]_hallii_group*, in cows at 21 days of gestation compared to non-pregnant cows. Additionally, notable decreases were observed in serum concentrations of metabolites, including bonactin, Pro-Hyp, PGF2α, and LTB4. These findings suggest that components of the vaginal microbiota, such as the *[Eubacterium]_hallii_group*, along with serum metabolites like bonactin, Pro-Hyp, PGF2α, and LTB4, may serve as potential biomarkers for early gestation, specifically at 21 days, in cows. Further research is required to validate and expand the screening of these biomarkers at 21 days of gestation. As research progresses, these biomarkers hold promise for the early detection of pregnancy in cows.

## Figures and Tables

**Table 1:** A comparison of alpha diversity parameters.

Groups	Chao1	Shannon	Simpson
NP	1776.93 ± 215.56	8.52 ± 0.62	0.98 ± 0.02
P	1706.72 ± 209.55	7.69 ± 2.47	0.93 ± 0.11
*p*-value	0.71	0.60	0.48

Note: Values are expressed as mean ± standard deviation.

**Table 2:** Significant changes in serum metabolites between the pregnant and non-pregnant groups.

Number	Metabolite	*m*/*z*	RT	VIP	*p*-Value	Fold Change	AUC
1	Bonactin	399.22	30.396	1.472	0.007	3.053	0.972
2	2-deoxy-d-glucose	187.048	262.733	1.544	0.047	2.438	0.806
3	2-amino-2-methyl-1-propanol	90.091	594.255	3.729	0.006	1.562	0.833
4	Cabergoline	452.314	218.311	3.398	0.049	1.246	0.917
5	Guanidinoethyl sulfonate	166.018	207.754	1.752	0.042	1.2	0.833
6	3,3′-dimethoxybenzidine	245.15	328.868	1.606	0.039	1.18	0.861
7	3-pyridinecarboxaldehyde	108.055	366.259	1.073	0.03	1.175	0.889
8	Phosphoric acid	96.96	445.631	3.755	0.019	0.927	0.861
9	Piperidine	86.097	290.788	3.835	0.039	0.869	0.806
10	1-o-hexadecyl-2-o-(4z,7z,10z,13z,16z,19z-docosahexaenoyl) -sn-glyceryl-3-phosphorylcholine	792.59	38.166	1.688	0.029	0.828	0.861
11	Pc 38:4	868.606	167.263	1.316	0.038	0.798	0.861
12	Propionylcarnitine	218.139	307.294	7.282	0.005	0.629	0.944
13	D-2-aminobutyric acid	104.071	504.695	3.585	0.044	0.574	0.806
14	Leukotriene b4	339.232	30.839	2.312	0.019	0.558	0.917
15	Pro-hyp	229.118	448.612	3.526	0	0.348	1
16	2-aminophenol	110.071	446.875	3.163	0.014	0.319	0.806

Note: Sample size: NP = 6; P = 6. NP, non-pregnant group; P, pregnant group. MS detection mode: NEG, negative; POS, positive.

## Data Availability

The raw data supporting the conclusions of this article will be made available by the authors upon request.

## Supplementary Materials

The following supporting information can be downloaded at: https://www.mdpi.com/article/10.3390/metabo14090469/s1, Figure S1: Multivariate statistical analysis of serum metabolites.(A) Positive ion mode serum metabolic profiles; (B) Negative ion mode serum metabolic profiles; (C) Positive ion mode PCA scoring plots; (D) Negative ion mode PCA scoring plots; (E) Positive ion mode OPLS-DA scoring plots; (F) Negative ion mode OPLS-DA scoring plots; (G) Positive ion mode OPLS-DA permutation test plots; (H) Negative ion mode OPLS-DA replacement test plot.
