# Supplementary material for: Combining the Vaginal Microbiome and Serum Metabolome to Screen for Potential Biomarkers of Early Pregnancy in Cows
Source: Metabolites. 2024 Aug 26;14(9):469. doi: 10.3390/metabo14090469 (PMC11434538; doi:10.3390/metabo14090469)
Supplement: Supplementary file 1 [file metabolites-14-00469-s001.zip › metabolites-3141342-supplementary.pdf]

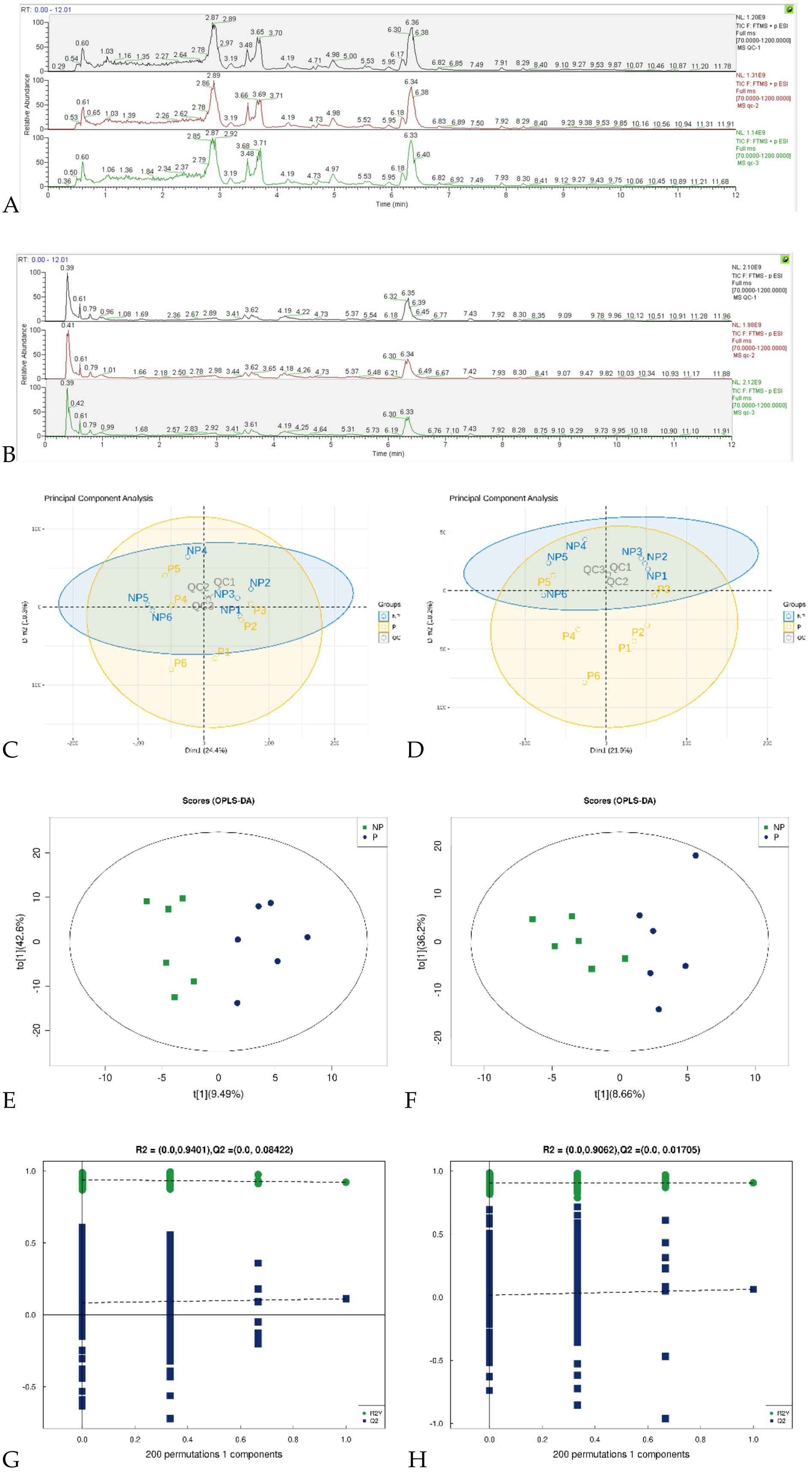
A
RT: 0.00 - 12.01
Relative Abundance
Time (min)
NL: 1.20E9 TIC F: FTMS + p ESI Full ms [70.0000-1200.0000] MS QC-1
NL: 1.31E9 TIC F: FTMS + p ESI Full ms [70.0000-1200.0000] MS qc-2
NL: 1.14E9 TIC F: FTMS + p ESI Full ms [70.0000-1200.0000] MS qc-3
B
RT: 0.00 - 12.01
Relative Abundance
Time (min)
NL: 2.10E9 TIC F: FTMS - p ESI Full ms [70.0000-1200.0000] MS QC-1
NL: 1.98E9 TIC F: FTMS - p ESI Full ms [70.0000-1200.0000] MS qc-2
NL: 2.12E9 TIC F: FTMS - p ESI Full ms [70.0000-1200.0000] MS qc-3
C
Principal Component Analysis
Dim1 (24.4%)
Groups
D
Principal Component Analysis
Dim1 (21.9%)
Groups
E
Scores (OPLS-DA)
t[1](9.49%)
to[1](42.6%)
NP
P
F
Scores (OPLS-DA)
t[1](8.66%)
to[1](36.2%)
NP
P
G
R2 = (0.0,0.9401),Q2 =(0.0, 0.08422)
200 permutations 1 components
H
R2 = (0.0,0.9062),Q2 =(0.0, 0.01705)
200 permutations 1 components

Figure S1. Multivariate statistical analysis of serum metabolites.(A) Positive ion mode serum metabolic profiles; (B) Negative ion mode serum metabolic profiles; (C) Positive ion mode PCA scoring plots; (D) Negative ion mode PCA scoring plots; (E) Positive ion mode OPLS-DA scoring plots; (F) Negative ion mode OPLS-DA scoring plots; (G) Positive ion mode OPLS-DA permutation test plots; (H) Negative ion mode OPLS-DA replacement test plot.
